# Passive amyloid-β immunotherapy in Alzheimer’s disease: a multicellular clearance system beyond plaque removal

**DOI:** 10.1186/s13024-026-00972-y

**Published:** 2026-07-16

**Authors:** Xiaoni Zhan, Chenchen Liu, Changjiang Yu, Nils Lindblom, Tomas Deierborg, Asgeir Kobro-Flatmoen, Gunnar K. Gouras, Gehua Wen

**Affiliations:** 1 School of Forensic Medicine, China Medical University, Shenyang, Liaoning Province China; 2 Neural Plasticity and Repair Unit, Department of Experimental Medical Science, Lund University, Lund, Sweden; 3 Department of Neurology, Tongji Hospital, Tongji Medical College, Huazhong University of Science and Technology, Wuhan, China; 4 Department of Neurology, The Fourth Affiliated Hospital of China Medical University, Shenyang, Liaoning Province China; 5 Experimental Dementia Research Unit, Department of Experimental Medical Science, Lund University, Lund, Sweden; 6 Experimental Neuroinflammation Lab, Department of Experimental Medical Science, Lund University, Lund, Sweden; 7 Kavli Institute for Systems Neuroscience, Norwegian University of Science and Technology, Trondheim, Norway; 8 Norwegian Health Association Centre for Dementia Research, Norwegian University of Science and Technology (NTNU), Postbox, Trondheim, 8905, 7491 Norway

**Keywords:** Alzheimer's disease, Amyloid-β immunotherapy, Multicellular clearance network, Amyloid-related imaging abnormalities (ARIA), Cell-type-specific-interactions

## Abstract

Passive immunotherapy targeting amyloid-β (Aβ) has emerged as a major therapeutic strategy for Alzheimer’s disease (AD), yet its clinical benefits remain modest and are frequently accompanied by vascular adverse events such as amyloid-related imaging abnormalities (ARIA). While the removal of extracellular Aβ plaques is associated with therapeutic efficacy, accumulating evidence suggests that additional cellular and vascular mechanisms may also contribute to complementary therapeutic outcomes alongside plaque removal. Recent studies show that Aβ antibodies are broadly distributed within the brain and interact with multiple neural and immune cell populations, rather than being limited to Aβ plaques. These observations support an expanded view of passive Aβ immunotherapy as a multicellular coordinated clearance process. Aβ antibodies engage diverse cellular and anatomical compartments, including neurons, glial cells, perivascular macrophages, peripheral immune cells, and meningeal lymphatic pathways, thereby influencing Aβ dynamics across intracellular and extracellular pools. Within this framework, therapeutic outcomes are influenced not only by plaque clearance but also by interactions between Aβ antibodies and cellular and anatomical compartments that regulate Aβ clearance and treatment-associated vascular response. This perspective may help explain variability in clinical efficacy and the emergence of vascular side effects, while also providing additional considerations for optimizing Aβ antibody design and therapeutic strategies.

## Introduction

Alzheimer’s disease (AD) is characterized by extracellular amyloid-β (Aβ) deposition, intracellular tau pathology, and interconnected neuroinflammatory and neurovascular alterations that collectively contribute to disease progression and cognitive decline [1, 2]. Therapeutic strategies targeting both Aβ and tau pathology have shown substantial advances in recent years [3–5]. Among these approaches, passive Aβ immunotherapy has achieved advanced clinical translation to date, with several monoclonal antibodies, including aducanumab, lecanemab, and donanemab, demonstrating that Aβ immunotherapy can robustly reduce plaque burden and modestly slow cognitive decline in patients with early AD [6–8]. However, despite substantial reductions in plaque burden, the associated clinical benefits remain relatively limited and are frequently accompanied by vascular adverse events such as amyloid-related imaging abnormalities (ARIA). Although amyloid plaque reduction is considered an important therapeutic correlate, the relationship between plaque clearance and cognitive improvement remains incompletely understood [9, 10]. Moreover, increasing evidence indicates that Aβ antibodies are not limited to extracellular plaques but distribute broadly across neuronal, glial, and vascular compartments [11]. These observations support an expanded view of Aβ immunotherapy that includes multicellular coordinated processes in addition to plaque clearance. Such findings raise the possibility that therapeutic outcomes may be shaped not only by plaque removal, but also by interactions among neural, immune, and vascular compartments. Accordingly, an important question is how these interactions influence therapeutic efficacy and safety in the brain.

Neurons, glial cells, and perivascular or peripheral immune populations may together participate in regulating Aβ clearance within the brain. Aβ antibodies can be internalized by neurons, where they may influence intraneuronal Aβ dynamics [11, 12], suggesting that therapeutic effects extend beyond extracellular plaque removal. In parallel, microglia respond to antibody–Aβ complexes through Fc receptor–dependent and independent mechanisms, mediating local clearance but also contributing to inflammatory responses [13–18]. Astrocytes also exhibit metabolic and functional alterations during immunotherapy [19, 20]. Moreover, perivascular macrophages (PVMs) and meningeal lymphatic vessels (mLVs), key components of neurovascular and fluid-mediated clearance pathways, have emerged as potential modulators of Aβ antibody distribution and efficacy [21–23].

Viewing Aβ immunotherapy as a multicellular coordinated clearance process provides a mechanistic framework for understanding variability in clinical efficacy and the occurrence of vascular adverse events. This perspective highlights the contributions of neurons, glial cells, perivascular macrophages, peripheral immune cells, and meningeal lymphatic vessels in shaping Aβ dynamics and therapeutic responses, emphasizing that the effects of Aβ antibodies extend beyond direct plaque removal to involve multicellular and neurovascular mechanisms that influence both treatment efficacy and safety.

## From early development to mechanistic insights in Aβ immunotherapy

**Fig. 1:**
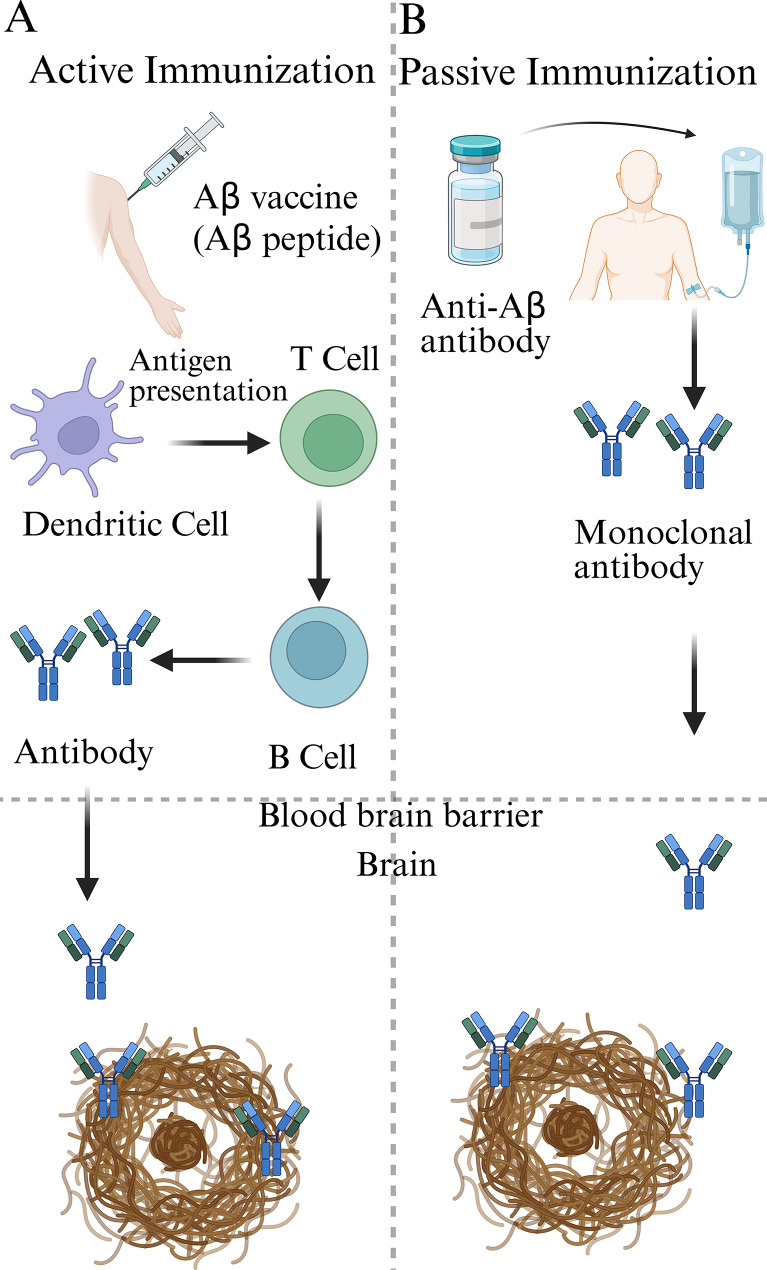
Comparison of active and passive Aβ immunization strategies in AD. (**A**) Active immunization: Administration of an Aβ-based vaccine leads to antigen presentation by dendritic cells to T cells, which subsequently activate B cells. Activated B cells produce anti-Aβ antibodies that cross the blood–brain barrier (BBB) and bind to amyloid plaques in the brain. (**B**) Passive immunization: Direct administration of exogenous anti-Aβ monoclonal antibodies, which cross the BBB and bind to amyloid plaques

Aβ immunotherapy is generally classified into two types: active immunotherapy, which involves stimulating the immune system using Aβ-derived antigens, and passive immunotherapy, which consists of administering externally produced monoclonal antibodies designed to target different Aβ species, epitopes, or aggregation states [24]. Active immunotherapy is designed to induce a sustained endogenous antibody production in the host [24]. This approach can achieve relatively high antibody levels with only a limited number of administrations. By contrast, passive immunotherapy involves the direct delivery of exogenous antibodies, providing immediate but transient effects [25]. These two strategies have defined the early development of Aβ immunotherapy and have provided important mechanistic and translational insights. The fundamental distinctions between active and passive Aβ immunotherapy are summarized in Fig. 1.

Early studies established the principle that Aβ immunotherapy can promote Aβ clearance. In vitro experiments showed that monoclonal antibodies can inhibit Aβ fibrillization and disrupt preformed aggregates [26, 27], and subsequent in vivo studies revealed that Aβ42 immunization markedly reduced amyloid burden and gliosis in transgenic mouse models [28]. Active Aβ vaccination was also shown to attenuate plaque pathology and improve cognitive performance in APP/PS1 mice [29]. However, clinical translation revealed major limitations. The AN1792 vaccine induced antibody responses in patients [30, 31] but was terminated owing to aseptic meningoencephalitis [32]. Postmortem analyses confirmed substantial plaque clearance and microglial recruitment, together with persistent vascular amyloid deposition [32], indicating that amyloid removal can cause deleterious immune effects. Although subsequent vaccination strategies continued to show efficacy in animal models [33], safety concerns following early clinical trials prompted the development of next-generation active immunization approaches. Several of these vaccines have demonstrated encouraging safety and tolerability profiles in clinical studies [34, 35].

In parallel with the continued development of active immunization strategies, passive Aβ immunotherapy emerged as an important therapeutic strategy. Studies showed that chronic antibody administration robustly reduced parenchymal amyloid burden in AD mouse models [36]. To explain these effects, the peripheral sink hypothesis was proposed based on observations that antibody-mediated peripheral binding of Aβ was associated with marked increases in plasma Aβ levels [37]. However, subsequent studies indicated that Aβ antibodies can enter the brain parenchyma and bind Aβ without acutely promoting Aβ efflux, indicating that peripheral mechanisms alone are insufficient to fully explain amyloid reduction in vivo [38]. Meanwhile, preclinical investigations revealed that passive Aβ immunotherapy increases the risk of cerebral microhemorrhages associated with cerebral amyloid angiopathy (CAA), despite reductions in parenchymal plaques [39–41]. Early studies of both active and passive Aβ immunotherapy indicated that immune-based approaches can alter amyloid pathology, while also revealing limitations related to the vascular responses.

## Clinical development of passive Aβ immunotherapy

The clinical development of passive Aβ immunotherapy has involved multiple generations of monoclonal antibodies with differing target epitopes, pharmacological properties, and trial designs. Although many of these antibodies have achieved substantial reductions in amyloid burden, clinical benefits have remained modest and safety concerns, particularly ARIA, continue to limit their clinical impact. An overview of major antibody generations and their clinical characteristics is provided in Table 1.

**Table 1:** Summary of passive Aβ immunotherapy development

Generation	Antibody	Epitope/ Binding preference	Clinical findings	ARIA incidence	Clinical outcomes
First	Bapineuzumab (3D6)	N-terminal Aβ_1–5_;	PET confirmed plaque reduction; limited cognitive benefit; ApoE ε4 ↑ ARIA risk	High, especially ARIA-E	Development discontinued (2007–2014) [42]
	Solanezumab (m266)	Mid-domain Aβ_16–26_;	↑ Plasma & CSF Aβ; modest cognitive slowing in mild AD	Low	Phase III (NCT01900665) failed; discontinued [44–47]
Second	Crenezumab	Mid-domain Aβ_13−24_; preferential binding to soluble oligomeric Aβ;	No significant slowing of cognitive decline in Phase III trials	Low	Phase III trials failed to meet primary endpoints; development discontinued [213–215]
	Gantenerumab	N-terminal and Mid-domain Aβ_3–11_ and Aβ_18–27_;	Dose-dependent amyloid PET reduction	Moderate ↑ risk in ApoE ε4	No FDA approval [48–50, 216]
	Ponezumab	C-terminal selective for Aβ40	Safe, minimal ARIA; no cognitive/amyloid PET benefit	Minimal	Discontinued (insufficient efficacy) [51–54]
	Aducanumab	N-terminal Aβ_3−7_ Aggregated Aβ	Strong amyloid PET clearance	High ARIA (dose-dependent)	FDA accelerated approval (2021) [55–60]
	Lecanemab (mouse mAb158)	N-terminal Soluble protofibrils	Robust amyloid clearance; moderate slowing of cognitive decline.	ARIA-E: 12.6%, ARIA-H: 17.3%	FDA approval (2023); Clinical use ongoing [8, 62, 63, 65, 73, 76]
	Donanemab (mouse mE8-IgG2a)	N-terminal Aβ3pE-x Pyroglutamate-modified Aβ	Rapid and durable amyloid clearance; modest tau reduction; slowed decline.	ARIA-E: 20–24%, ARIA-H: 27–31%	FDA approval (2024); early use in MCI/mild AD [77–82]
Third	Remternetug	Aβ3pE-x Pyroglutamated Aβ	Selectivity, binding deposited plaque cores rather than soluble Aβ	Most ARIA events are mild, and manageable with MRI monitoring and dose adjustments.	Fast Track designation from the FDA, Currently in Phase 3 trials (NCT05463731) [91, 217]
	Trontinemab	Similar to the binding mode of gantenerumab	Designed with Brainshuttle technology to enhance penetration across the blood-brain barrier.	ARIA-E reported in < 5%	Currently in Phase 3 trials (NCT07169578) [93, 95]

First-generation passive Aβ immunotherapies provided evidence of amyloid target interaction in AD but did not translate into consistent clinical benefit. Bapineuzumab treatment reduced amyloid PET signal, consistent with decreased amyloid plaque burden, but was associated with a high incidence of ARIA, particularly vasogenic edema, while showing limited cognitive benefit in clinical trials [42]. Solanezumab, derived from the murine antibody m266 and designed to preferentially bind soluble Aβ species, altered plasma and cerebrospinal fluid (CSF) Aβ but did not improve cognitive or functional outcomes in phase III trials [43–47].

Second-generation antibodies were developed with distinct epitope and aggregation-state specificities to improve Aβ targeting and amyloid clearance. Several antibodies, including gantenerumab, achieved dose-dependent reductions in amyloid burden but showed limited cognitive benefit, while clinical use was often complicated by ARIA-related safety concerns [48–50]. In contrast, ponezumab, which selectively recognizes the C-terminus of Aβ40, exhibited limited plaque engagement and minimal ARIA liability, highlighting the mechanistic diversity among Aβ antibodies [51–54].

**Fig. 2:**
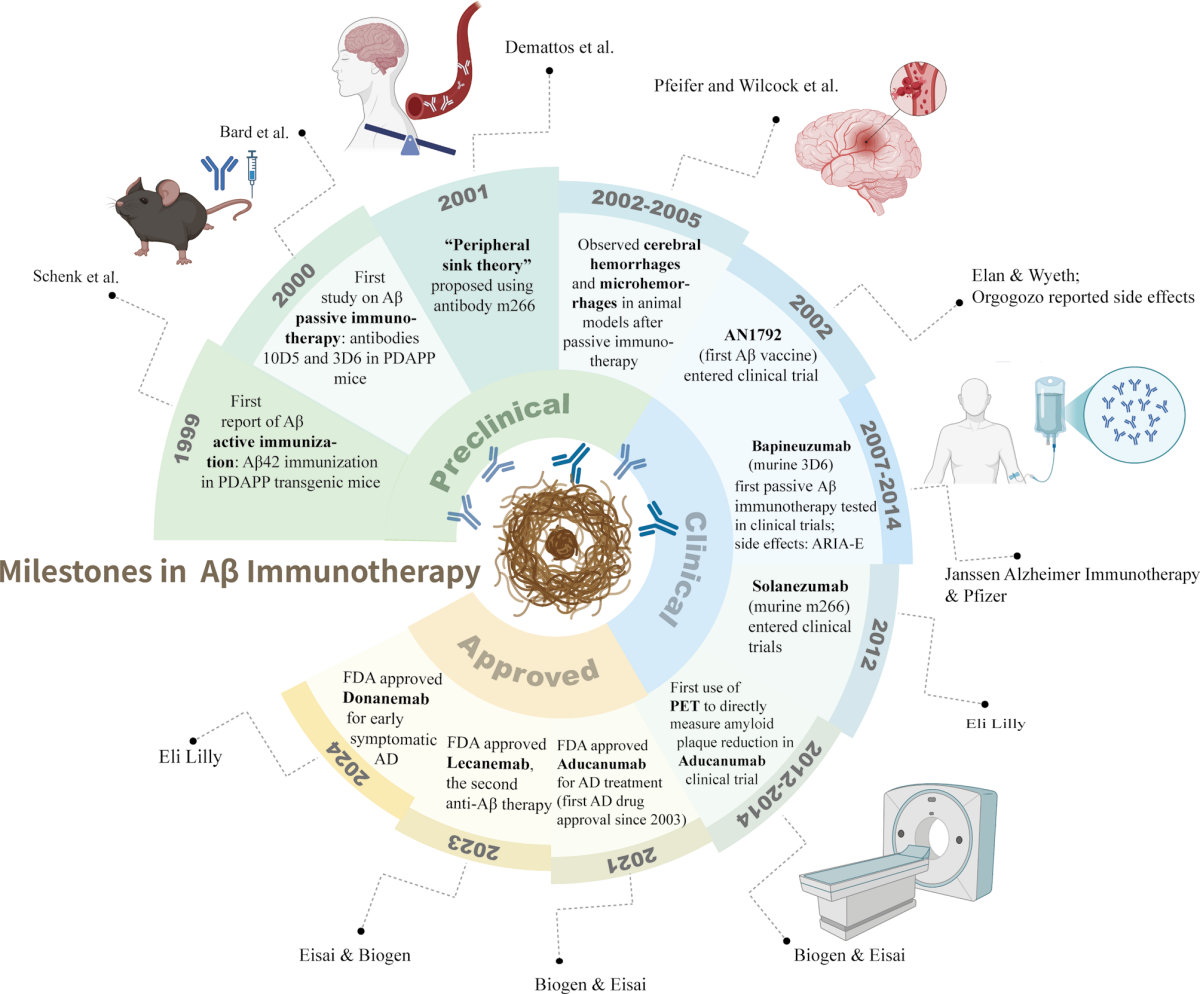
Milestones in Aβ immunotherapy. Key developments in Aβ immunotherapy for AD. Initial breakthroughs included active and passive Aβ immunization in transgenic mouse models (1999–2000), followed by the proposal of the “peripheral sink hypothesis” (2001) and recognition of treatment-associated cerebral microhemorrhages (2002–2005). Early clinical trials, such as AN1792, bapineuzumab, and solanezumab, revealed both clinical effects and safety concerns. A major advance came with the introduction of PET imaging to directly quantify amyloid plaque reduction in clinical studies, providing an objective biomarker to evaluate treatment efficacy. More recently, the FDA approvals of aducanumab, lecanemab, and donanemab have marked critical milestones, providing clinical evidence for disease modification by Aβ antibodies, although their long-term clinical benefits remain to be fully determined

Aducanumab represents a major advance by providing clear evidence of substantial plaque reduction and becoming the first Aβ antibody to receive FDA approval, although its approval remained controversial owing to inconsistent cognitive outcomes and dose-dependent safety concerns [7, 55–60]. Subsequent antibodies were engineered to recognize more specific Aβ conformations. Lecanemab, which preferentially targets soluble Aβ protofibrils, produced robust amyloid clearance and showed a modest slowing of cognitive decline in early AD [8, 61–76]. Donanemab, targeting pyroglutamate-modified Aβ species enriched in mature plaques, similarly achieved rapid plaque removal with modest clinical benefit, but was associated with a relatively high incidence of ARIA [77–82]. Subsequent studies employing titration dosing strategies, however, reported improved safety profiles with lower ARIA rates [83]. Further, we summarize the development of passive Aβ immunotherapy from early studies to recent clinical milestones, highlighting the advances and constraints that have contributed to the field (Fig. 2).

Despite substantial progress in Aβ antibody development, limited understanding of how therapeutic antibodies distribute within the brain remains an important challenge in passive Aβ immunotherapy. Boon et al. reported that amyloid plaque clearance in aducanumab-treated AD brains was most pronounced in superficial cortical layer I, whereas deeper cortical regions exhibited more limited plaque removal [84]. In addition, conventional Aβ antibodies exhibit limited penetration into deeper brain regions and preferential localization to CSF-associated and perivascular compartments, which may contribute to incomplete engagement of parenchymal amyloid pathology [85]. Together, these findings suggest that the delivery of conventional Aβ antibodies into the brain is constrained by the restrictive properties of the BBB, thereby limiting effective engagement of amyloid pathology within the CNS. To overcome these limitations, Pizzo et al. developed an antibody transport vehicle (ATV) platform that utilizes transferrin receptor (TfR)-mediated transcytosis to facilitate BBB transport and enhance antibody delivery into the brain parenchyma [85]. Unlike conventional monoclonal antibodies, which primarily exhibit limited entry through CSF and perivascular pathways, ATV-modified antibodies engage TfR expressed on brain capillary endothelial cells, enabling broader transport across the cerebral microvasculature into the brain parenchyma [85]. This modified delivery route resulted in substantially increased CNS exposure and more widespread plaque engagement throughout the brain in AD mouse models [85]. These advances have further accelerated the development of third-generation Aβ antibodies designed to improve brain delivery and therapeutic efficacy.

Third-generation Aβ antibodies, such as Remternetug and Trontinemab, have been developed to overcome limitations of earlier Aβ antibodies. Compared with first- and second-generation antibodies, these approaches place greater emphasis on optimizing pharmacokinetic properties, enhancing target engagement, and improving brain delivery. Like Donanemab, Remternetug has been designed to overcome some limitations associated with earlier anti-pyroglutamate antibodies, including antidrug antibody formation and infusion-related reactions, while also supporting alternative administration strategies such as subcutaneous delivery [86–88]. Phase I studies of Remternetug in healthy participants and individuals with AD demonstrated acceptable safety and dose-dependent plaque reduction, with the highest intravenous dose reducing amyloid burden below 24 centiloids (CL) within three months [89]. A reduction of brain amyloid burden to below 24 CL indicates that fibrillar Aβ levels, as measured by PET imaging, have been lowered to a range considered amyloid-negative or non-pathological, typically around 20–25 CL [90]. Building on these findings, the phase III TRAILRUNNER-ALZ1 study (NCT05463731) is evaluating intravenous and subcutaneous remternetug administration in approximately 600 patients with early symptomatic AD, with reduction of amyloid burden assessed by PET imaging as the primary endpoint [91]. In parallel, the larger phase III TRAILRUNNER-ALZ3 study is evaluating the effects of Remternetug on cognitive and functional outcomes in patients with early AD, providing critical evidence regarding the clinical efficacy of treatment beyond biomarker changes [92].

Unlike conventional Aβ antibodies, Trontinemab combines gantenerumab-derived Aβ binding with a BrainShuttle-mediated transferrin receptor 1 (TfR1) transport module, enabling receptor-mediated BBB transcytosis and enhanced brain delivery while preserving target specificity [93, 94]. In the Phase Ib/IIa trial, high-dose Trontinemab achieved rapid amyloid clearance, leading to amyloid negativity in over 80% of patients by week 28 [95]. These imaging findings were consistent with parallel declines in CSF and plasma tau biomarkers, including total tau and p-tau181/217 [95]. Trontinemab was well tolerated with ARIA-E reported in < 5% of cases [95]. Encouraged by these outcomes, Roche has scheduled two phase III studies that started in 2025 [96]. Trontinemab thus emerges as a promising approach to mitigate some of the limitations of existing Aβ antibodies, offering rapid amyloid clearance, although its clinical efficacy on cognition and disease progression has yet to be determined.

### Epitope specificity as an intrinsic property of therapeutic Aβ antibodies

**Fig. 3:**
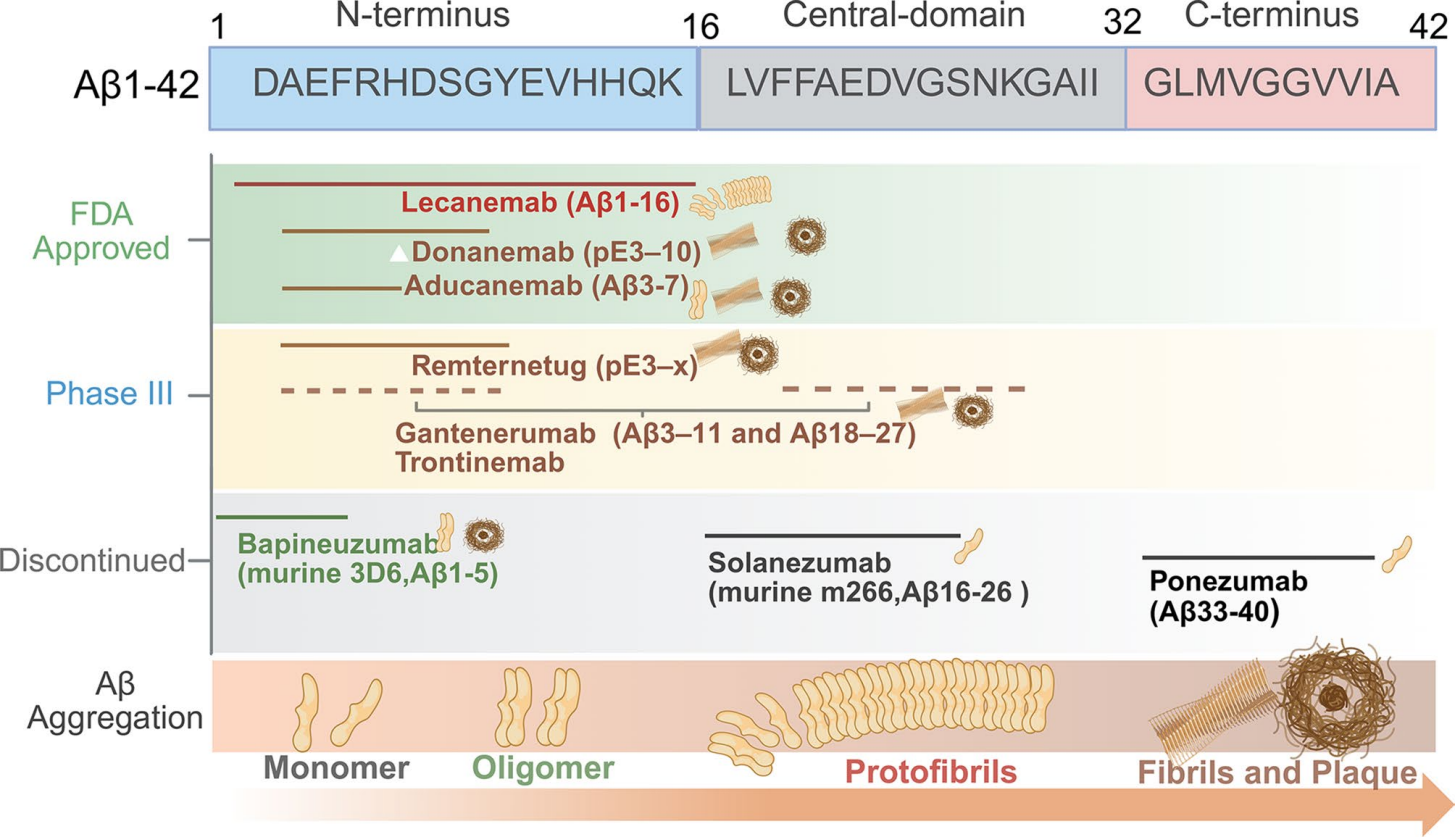
Epitope targeting of Aβ antibodies across Aβ aggregation states. Overview of the Aβ₁–₄₂ peptide sequence, highlighting the N-terminal, central-domain, and C-terminal domains, together with the approximate epitopes recognized by major Aβ antibodies. Antibodies are grouped according to their clinical development status. FDA-approved antibodies include lecanemab, which targets an N-terminal epitope (Aβ1---16) with preference for soluble protofibrils, donanemab, which selectively recognizes pyroglutamate-modified Aβ (AβpE3) enriched in mature plaques, and aducanumab, which binds aggregated N-terminal Aβ (Aβ3–7). Investigational antibodies shown in phase III development include Remternetug, which recognizes pyroglutamate-modified Aβ (pE3–x), and Trontinemab, a next-generation gantenerumab-derived antibody engineered to enhance brain delivery. Gantenerumab, which recognizes N-terminal and central Aβ epitopes (Aβ3–11 and Aβ18–27), completed phase III clinical testing but did not demonstrate clinical benefit and has not been advanced further. Discontinued antibodies, including Bapineuzumab (Aβ1–5), Solanezumab (Aβ16–26), and Ponezumab (Aβ33–40), are shown for comparison. The lower section illustrates the progression of Aβ aggregation states, from monomers and oligomers to protofibrils and fibrillar plaques, highlighting how epitope selection links individual antibodies to distinct Aβ forms and clinical outcomes

The epitope targeted by Aβ antibodies defines which Aβ conformations are recognized and therefore strongly influences biological activity. Antibodies directed against the N-terminal region of Aβ preferentially bind aggregated and fibrillar species that remain accessible within amyloid plaques, facilitating plaque binding and microglia-associated clearance [36, 78, 97, 98]. By contrast, antibodies targeting central or C-terminal epitopes show limited plaque binding in vivo and instead preferentially interact with soluble Aβ species, including those in the circulation, thereby influencing peripheral Aβ dynamics without directly promoting plaque removal [99]. Consistent with these mechanistic differences, preclinical studies in AD mouse models have shown that N-terminal antibodies produce more robust reductions in plaque burden than antibodies targeting other regions of Aβ [92, 93]. These findings have informed clinical development strategies and contributed to the predominance of N-terminal antibodies in human trials. Indeed, clinical imaging studies indicated that such antibodies can induce substantial reductions in PET-detectable amyloid burden, ranging from partial decreases to near-complete clearance depending on antibody properties and dosing strategy [100]. Key features of epitope specificity, clinical outcomes, and safety profiles across antibody generations are summarized in Fig. 3.

## Cellular effects of passive Aβ immunotherapy in the CNS

### Neuron

#### Intraneuronal Aβ and neuronal dysfunction in AD

Growing evidence indicates that the accumulation of intraneuronal Aβ (iAβ) is an early contributor to neuronal dysfunction in AD. Importantly, iAβ buildup precedes the formation of extracellular plaques in both humans and animal models [101]. In 1993, it was first reported that Aβ can be found within cells. Using retinoic acid-differentiated NT2 neuronal cells (NT2N), a human neuronal model, Aβ was detected under constitutive intracellular conditions [102]. Meanwhile, studies on human subjects showed that in individuals with Down syndrome, elevated levels of Aβ42, but not Aβ40, were detectable before plaque formation [103]. And work on AD subjects revealed that cognitive decline correlates more closely with soluble Aβ levels than with plaque burden [104]. Early studies by Gouras and colleagues identified pronounced, region-specific iAβ accumulation in individuals with Down syndrome and MCI, particularly within pyramidal neurons of the hippocampus and entorhinal cortex, regions known to be affected early in AD [101]. These findings suggested that increased levels or altered forms of iAβ42 are a key early pathological event in humans. Mouse or rat models consistently support these observations. 3xTg-AD mice accumulate iAβ at 3–4 months, preceding plaques at 6 months [105], and 5xFAD mice display iAβ by 1.5 months, just before plaque deposition at 2 months [106]. The same is the case for the McGill-R-Thy-APP rat model, where increased iAβ is present by postnatal day 15, while the first plaques do not appear before 6–9 months [107]. These models consistently place iAβ42 upstream of extracellular amyloid deposition.

Importantly, iAβ accumulation is not uniform, but instead preferentially affects selectively vulnerable neuronal populations. Neuropathological studies have shown that iAβ appears in AD vulnerable neurons within regions such as the hippocampus and entorhinal cortex prior to extensive plaque deposition and neuronal loss, suggesting that iAβ may represent an early pathological event linked to neuronal susceptibility to degeneration [101, 108, 109]. Recent work has further supported this concept by demonstrating that iAβ accumulation specifically marks neuronal populations that are selectively lost during disease progression. Using a combination of imaging mass cytometry and single-nucleus RNA sequencing in the human middle temporal gyrus cortex, Caramello and colleagues identified several neuronal subtypes that undergo preferential degeneration in AD, particularly RAR-related orphan receptor beta (RORB)-positive and glutamate decarboxylase 1 (GAD1)-positive neuronal populations, which have previously been implicated as selectively vulnerable neuronal classes [110]. Spatial analyses further showed that activated cluster of differentiation 68 (CD68) positive microglia were preferentially located near neuronal subtypes accumulating iAβ, whereas reactive Glial Fibrillary Acidic Protein (GFAP)-positive astrocytes were associated with neuronal populations that remained relatively preserved even at later disease stages [110]. These findings suggest that local inflammatory responses and neuron-glia interactions may contribute to the selective degeneration of specific neuronal populations. A network-based analysis integrating neuron-specific transcriptomics with human genetic datasets identified molecular programs that distinguish neuronal populations vulnerable to AD from relatively resistant ones. Vulnerable neurons, particularly excitatory neurons in the entorhinal cortex and hippocampus, showed enrichment of pathways related to amyloid precursor protein processing, endosomal trafficking, and synaptic activity [111]. Because APP processing and endosomal dysfunction are closely linked to Aβ generation and intracellular accumulation, these intrinsic molecular features may create a cellular environment that promotes iAβ buildup in selectively vulnerable neuronal populations [111]. Neuronal activity may further contribute to the preferential accumulation of Aβ within vulnerable circuits. For example, sensory deprivation induced by unilateral whisker trimming reduces neuronal activity in the corresponding barrel cortex and leads to a marked increase in iAβ accumulation, indicating that synaptic activity modulates iAβ generation [112]. At the same time, brain regions exhibiting higher baseline neuronal activity show greater amyloid deposition in AD mouse models, suggesting that network activity influences the regional distribution of amyloid pathology [109, 113].

These findings suggest that the selective accumulation of iAβ may have important pathological consequences for neuronal function. Accumulating evidence is consistent with the notion that iAβ may play an active role in neuronal dysfunction [12]. Oddo et al. (2005) showed that at four months, the stage when plaques are absent, 3xTg-AD mice already display Aβ-immunoreactive neurons in the cortex, hippocampus, and amygdala associated with synaptic dysfunction [114]. ArcAβ mice, expressing human APP with Swedish and Arctic mutations, show significant spatial learning and memory deficits as assessed by the Morris water maze, Y-maze, and active avoidance tests by six months of age, despite the absence of extracellular amyloid plaques; at this stage, iAβ deposits are observed in the cortex and hippocampus [115]. Another example is the case of the abovementioned McGill-R-Thy-APP rats. Prior to plaque deposition, they show subtle neuronal metabolic impairments [116].

These observations suggest that targeting iAβ may represent a potential strategy to ameliorate early neuronal dysfunction. Passive Aβ immunotherapy has demonstrated efficacy in reversing cognitive impairments in AD mouse models. In aged PDAPP mice, treatment with the monoclonal antibody m266 led to a rapid improvement in performance of the object recognition and holeboard tasks, even though total brain Aβ burden remained largely unchanged [117]. Similarly, 24-month-old PDAPP mice treated with m266 performed comparably to 8-month-old wild-type mice in object recognition tests [117]. These results indicate that cognitive improvements may primarily result from targeting soluble, toxic Aβ oligomers rather than reducing plaques. Meanwhile, there is evidence that intra- and extracellular Aβ pools are dynamically interrelated. Oddo et al. showed that a single intrahippocampal injection of the Aβ antibody 1560 reduced extracellular Aβ within 12 h, followed by decreased iAβ three days later [118]. Notably, once Aβ antibody levels declined, iAβ pathology reappeared before plaques reaccumulated, with an inverse correlation between the two pools in 3xTg-AD mice [118]. These findings highlight that clearance of one compartment can influence the other, pointing to the importance of considering both pools in therapeutic strategies. This interplay between intra- and extracellular Aβ further suggests that the effects of Aβ antibodies are not restricted to extracellular plaques but may also involve modulation of iAβ dynamics.

Consistent with this idea, both in vitro and in vivo studies indicate that Aβ immunotherapy can deplete iAβ. Tampellini et al. showed that internalized Aβ antibodies 6E10 and 4G8 reduce iAβ and rescue synapses in AD transgenic primary neurons [119]. In preclinical studies targeting intracellular Aβ, Oddo et al. showed that a single injection of antibodies 1560 or 4G8 into the hippocampus of 12-month-old 3xTg-AD mice not only reduced plaques but also cleared iAβ near the injection site, concurrently lowering early tau pathology [120]. In younger 3xTg-AD mice, intracerebroventricular injection of antibody 1560 led to an 86% reduction of iAβ in the hippocampus and fully rescued memory deficits within seven days [114]. However, once iAβ reaccumulated, cognitive impairments re-emerged [114]. In line with these observations, recent in vivo work shows that unilateral intrahippocampal injection of the Aβ antibody 6E10 leads to a marked reduction of iAβ within three days [11]. These findings highlight iAβ as a key pathological species and show that antibody effects extend beyond extracellular plaques to iAβ. Importantly, despite growing recognition of iAβ as a pathogenic species, no passive Aβ antibodies have been specifically engineered to selectively target neuronal intracellular Aβ. Current evidence derives primarily from studies showing that conventionally developed Aβ antibodies can undergo neuronal internalization and subsequently reduce iAβ levels. These findings suggest that neuronal uptake of Aβ antibodies may contribute to therapeutic efficacy. More broadly, they raise the possibility that future antibody engineering strategies could be designed to enhance neuronal entry and intracellular target engagement, thereby enabling more effective clearance of pathogenic iAβ species.

Emerging evidence further suggests that Aβ antibodies may directly undergo neuronal internalization and intracellular trafficking. Tampellini et al. showed that Aβ antibodies bind the extracellular Aβ domain of cell-surface APP and undergo dynamin-dependent internalization together with APP [119]. Internalized antibody–APP complexes subsequently traffic through endosomal–lysosomal compartments, and inhibition of lysosomal function prevents antibody-mediated reductions in intracellular Aβ, suggesting that endosomal–lysosomal degradation contributes to neuronal iAβ clearance [119]. These observations suggest that intracellular antibody trafficking may represent an important component of passive Aβ immunotherapy beyond extracellular plaque clearance. Interestingly, studies in tau immunotherapy have begun to establish more detailed intracellular antibody-processing mechanisms that may provide important mechanistic insights. Congdon et al. indicated that anti-tau antibodies can enter neurons through low-affinity Fcγ receptor-mediated endocytosis and traffic through the endosomal–lysosomal system, contributing to reductions in intracellular tau pathology [121]. Similarly, Collin et al. showed that anti-tau/pS422 antibodies are internalized by neurons and localize to endosomal and lysosomal compartments, where intracellular antibody uptake was associated with reduced tau accumulation and slowed pathological progression in a tau transgenic mouse model [122]. Notably, Gaikwad et al. reported that intranasally delivered anti-tau antibodies can directly target neuronal intracellular tau aggregates and promote their degradation through the intracellular Fc receptor tripartite motif-containing protein 21 (TRIM21), which mediates antibody-dependent intracellular protein degradation [123]. In contrast, although Aβ antibodies are internalized by neurons and can reduce iAβ burden, whether shared intracellular processing mechanisms, such as TRIM21-dependent degradation pathways, participate in neuronal iAβ clearance during passive Aβ immunotherapy remains incompletely understood and warrants further investigation.

Specifically, while preclinical studies consistently show that reducing iAβ ameliorates cognitive deficits, no clinical trial has directly assessed effects on iAβ in AD patients. This gap likely reflects current methodological limitations, as existing imaging approaches are unable to resolve iAβ in AD patients, and postmortem analyses of patients treated with Aβ antibodies remain limited. Moreover, clinical studies have largely focused on plaque clearance and cognitive outcomes as primary endpoints, yet cognitive benefits may not be fully explained by plaque removal alone, suggesting that current assessments may overlook intraneuronal effects of Aβ antibodies. This raises the possibility that part of the clinical benefits of Aβ antibodies may stem from their ability to lower iAβ. If so, therapeutic efficacy may depend not only on overall amyloid reduction but also on effective targeting of iAβ. Enhancing neuronal penetration to target intracellular pools could therefore represent a promising strategy for future Aβ antibody development aimed at maximizing cognitive benefits.

### Microglia

#### Microglial activation in Aβ immunotherapy

Microglia are the resident immune cells of the CNS and take part in tissue surveillance, removal of damaged neurons, misfolded proteins, and debris, as well as synaptic remodeling [124, 125]. In AD, Aβ aggregation rapidly recruits microglia to sites of plaque deposition, where they initially exert protective effects by phagocytosing Aβ aggregates [126–129]. With prolonged exposure to Aβ and sustained inflammatory signaling, microglia shift toward a dysfunctional state marked by cellular hypertrophy, reduced phagocytic capacity, and persistent release of pro-inflammatory cytokines [69, 129, 130]. This dysregulated activation contributes to neuronal injury and is associated with altered Aβ dynamics [131, 132]. This functional shift is accompanied by distinct transcriptional reprogramming in microglia. Transcriptomic analyses have shown that microglia responding to Aβ deposition transition into disease-associated states [133, 134]. These states are characterized by upregulation of genes, such as Triggering receptor expressed on myeloid cells 2 (TREM2), ApoE, Cystatin F (Cst7), C-Type Lectin Domain Family 7 Member A (Clec7a), and Integrin alpha X (Itgax), which are associated with phagocytic activity, lipid metabolism, and immune activation [133, 135]. Although early microglial activation supports Aβ clearance and neuroprotection, sustained activation amplifies neuroinflammation and accelerates neurodegenerative processes [136].

Microglial state changes are closely linked to their roles in Aβ deposition and clearance. Accumulating evidence implies that microglia contribute to early amyloid plaque seeding and subsequent plaque dynamics, rather than merely responding to existing Aβ deposits. In young APP^*NL−G−F*^ mice, microglial depletion reduced Aβ plaque burden and neuritic dystrophy, suggesting a role for microglia in early plaque seeding and growth [134]. These findings emphasize the stage-dependent functions of microglia during disease progression. Microglial activation is also a key component of the mechanism underlying Aβ immunotherapy. Pharmacological suppression of microglial activity with dexamethasone abolishes Aβ antibody-mediated removal of plaques [40]. In transgenic AD mouse models, treatment with aducanumab increases the proportion of activated microglia and induces a transcriptional program enriched for genes involved in complement signaling (C1qa, C1qb, C1qc), antigen presentation (H2-D1, H2-K1, B2m, Lgmn), and lysosomal function (Ctsh, Ctsz, Ctss) [16]. Together, these findings suggest that microglial activation is not uniformly protective or detrimental but instead exerts distinct effects on Aβ pathology and neurodegeneration.

#### FcγR–dependent and independent mechanisms of antibody-mediated Aβ clearance

Previous studies of Aβ vaccination already revealed a close association between plaque clearance and microglial activation surrounding residual amyloid deposits, implicating microglia as an important mediator of antibody-driven Aβ removal [28]. Building on these observations, Wilcock et al. showed that injection of an anti-Aβ IgG1 antibody targeting Aβ₁–₁₆ into the frontal cortex and hippocampus of Tg2576 mice resulted in a marked reduction in amyloid burden [137]. Importantly, this work suggested that antibody-mediated clearance involves two temporally distinct phases: an early phase (4–24 h) characterized by the removal of diffuse Aβ deposits that occurs largely independently of microglial activation, followed by a later phase (1–3 d) during which compact plaques are eliminated in association with pronounced microglial recruitment and activation [137]. Subsequent studies by the same group provided evidence that Fragment Crystallizable Gamma Receptors (FcγR) signaling is critical for the removal of compact fibrillar plaques [40]. Complementary ex vivo experiments further supported this mechanism. In primary microglia cultured on unfixed brain sections from PDAPP mice or human AD tissue, 10D5 treatment led to near-complete amyloid clearance and accumulation of Aβ-containing phagocytic vesicles, suggesting FcγR-dependent uptake [36]. Notably, this plaque-clearing effect required an intact Fc domain: while F(ab′)₂ fragments of the 3D6 antibody maintained full binding to Aβ plaques, they failed to induce microglial phagocytosis, whereas Fc receptor blockade abolished phagocytosis triggered by the intact antibody [36]. These preclinical observations are reflected in clinical studies of Aβ immunotherapy. Aducanumab has been proposed to promote plaque clearance by recruiting Iba1-positive microglia and enhancing Fc-dependent phagocytosis [7]. More recently, research from De Strooper’s lab indicated that the therapeutic efficacy of lecanemab depends on functional microglia and intact Fc-mediated effector mechanisms. In a human microglia xenograft mouse model, lecanemab markedly reduced amyloid pathology and Lysosome-Associated Membrane Protein (LAMP)+ dystrophies, but only in the presence of microglia expressing functional Fc variants [138].

Meanwhile, accumulating evidence further suggests that Fcγ receptor engagement may exert both beneficial and detrimental effects during Aβ immunotherapy. While Fc-mediated microglial activation promotes plaque phagocytosis, excessive Fc effector activity may also contribute to neuroinflammatory responses and synaptic injury [139]. Sun et al. indicated that Fc effector function of Aβ antibodies induces microglia-dependent synapse loss and cognitive deficits in AD mouse models, highlighting the potential neurotoxic consequences of excessive FcγR activation [139]. These findings suggest that Fc-mediated immune activation requires careful balance during passive Aβ immunotherapy, as excessive FcγR signaling may compromise the beneficial effects of amyloid clearance by promoting neuroinflammatory and synaptotoxic responses. These observations have stimulated interest in engineering antibody Fc domains to preserve therapeutic efficacy while minimizing excessive immune activation and vascular toxicity.

Importantly, the magnitude and nature of Fcγ receptor engagement are influenced by the Immunoglobulin G (IgG) isotype of therapeutic Aβ antibodies [140, 141]. Consistent with this concept, therapeutic Aβ antibodies used in clinical trials differ substantially in IgG isotype and Fc effector properties. Aducanumab and lecanemab are human IgG1 antibodies capable of engaging Fcγ receptors and promoting microglia-mediated plaque phagocytosis [7, 8], whereas crenezumab (MABT5102A, RG7412) was engineered on an IgG4 backbone with reduced effector function [139]. While this design may limit inflammatory activation and vascular adverse effects, reduced Fcγ receptor engagement may also attenuate microglia-dependent plaque clearance [139]. These observations suggest that Fc effector function represents a critical determinant of the balance between plaque clearance efficacy and inflammatory toxicity during passive Aβ immunotherapy. Consequently, increasing attention has been directed toward engineering antibody Fc domains to optimize this balance while preserving therapeutic efficacy.

Consistent with this emerging concept, engineered Fc variants have recently been incorporated into brain-penetrant antibody platforms. Pizzo et al. developed a transferrin receptor-targeted anti-Aβ antibody containing a cisLALA Fc mutation designed to reduce Fcγ receptor effector function while enhancing BBB transport [85]. This engineered approach increased brain delivery while mitigating ARIA-related vascular pathology in AD mouse models [85], highlighting the potential of Fc engineering strategies to improve the therapeutic safety and efficacy of Aβ antibodies.

Despite the substantial evidence supporting FcγR-mediated microglial clearance, several studies suggest that FcγR signaling is not absolutely required for antibody-induced amyloid reduction. Das et al. showed that Aβ immunization effectively reduced amyloid deposition in APP Tg2576 × FcRγ⁻/⁻ mice, indicating that FcγR signaling is dispensable under certain conditions [18]. Similarly, Hyman and colleagues reported that F(ab′)₂ fragments of Aβ antibodies lacking the Fc domain were still capable of reducing plaque burden in vivo [17]. These findings suggest that direct antibody–Aβ interactions alone can contribute to plaque removal. In addition to Fc-independent mechanisms, complement activation represents another important pathway. Fibrillar Aβ can activate the classical complement cascade, leading to C1q deposition and facilitating microglial clearance of antibody–Aβ complexes, particularly under conditions in which antibody concentrations are insufficient to drive efficient FcγR-mediated phagocytosis [142–144]. Taken together, these findings suggest that Aβ immunotherapy engages multiple, distinct but interconnected mechanisms, including FcγR-dependent microglial activation, complement-mediated clearance, and direct antibody–Aβ interactions. The relative contribution of these pathways may vary across antibodies and disease stages, indicating that antibody efficacy cannot be attributed to a single mechanism of action.

### Astrocytes

Astrocytes participate in both the clearance and accumulation of Aβ in AD. Early in the disease, astrocytes contribute to Aβ degradation, whereas with ageing and disease progression, impaired lysosomal function can lead to intracellular Aβ accumulation and secondary release, potentially promoting plaque growth [145–152]. Relatively few studies have directly examined how Aβ immunotherapy affects astrocytes. Available evidence suggests that astrocytes can participate in antibody-mediated Aβ uptake but have limited capacity to degrade the internalized material. Disruption of astrocytic Aβ uptake and degradation impairs brain Aβ clearance, as knockdown of low-density lipoprotein receptor-related protein 1 (LRP1) in astrocytes reduces Aβ uptake and degradation, for example, astrocyte-specific LRP1 deletion in APP/PS1 mice accelerates Aβ accumulation and plaque deposition without altering Aβ production [153]. Astrocytes can efficiently internalize Aβ protofibrils, yet often accumulate rather than degrade them, raising the possibility that they act as transient storage sites for Aβ [154]. Whether this process can be modified by Aβ antibodies remains unclear. The Aβ protofibril–selective antibody mAb158 (the murine precursor of lecanemab) prevents intracellular accumulation of Aβ in astrocytes when present during Aβ exposure, suggesting that antibody binding alters astrocytic handling of Aβ and limits its intracellular retention, with downstream protective effects on neurons [19].

In addition, recent in vivo observations further suggest that astrocytes may participate in local responses to antibody-bound amyloid pathology. Our recent study indicated that administration of the Aβ antibody 6E10 in AD mouse models revealed robust antibody localization to amyloid plaques, which were frequently surrounded by activated GFAP-positive astrocytes [11]. Notably, partial colocalization between 6E10 and plaque-associated astrocytes was observed, suggesting that astrocytes may interact with antibody-bound Aβ deposits within the plaque microenvironment. Although the functional significance of these interactions remains unclear, these findings raise the possibility that astrocytes contribute to local cellular responses during passive Aβ immunotherapy and warrant further investigation. Together, these findings suggest that astrocytes may participate in the handling of antibody–Aβ complexes and local responses to antibody-bound amyloid pathology during passive Aβ immunotherapy. However, the precise contribution of astrocytes to antibody-mediated amyloid clearance remains incompletely understood. Nevertheless, their intrinsic capacity to internalize Aβ has raised interest in whether this function can be therapeutically enhanced.

Accordingly, recent studies have begun exploring engineered astrocyte-based approaches designed to augment Aβ uptake and clearance within the CNS. Chen et al. developed chimeric antigen receptor astrocytes (CAR-As) engineered to recognize Aβ pathology, representing an experimental glial-based immunotherapeutic strategy for AD [155]. In contrast to conventional passive immunotherapy, this approach aims to enhance the intrinsic capacity of astrocytes to interact with and internalize Aβ aggregates within the CNS. In AD mouse models, CAR-As exhibited enhanced Aβ uptake and were associated with reductions in amyloid plaque burden, while showing relatively limited inflammatory activation compared with some microglia-based approaches [155]. Although still at an early experimental stage, these findings suggest that engineered astrocytes may represent a potential strategy for improving local Aβ handling within the brain. Nevertheless, direct evidence demonstrating that astrocytes serve as major effector cells during conventional passive Aβ immunotherapy remains limited, and their contribution may primarily involve modulation of Aβ redistribution, intracellular retention, and local handling of antibody–Aβ complexes rather than large-scale plaque clearance.

### Oligodendrocytes (OLs)

Oligodendrocytes (OLs) are specialized glial cells responsible for generating the multilayered, lipid-rich myelin sheath in the CNS, enabling rapid saltatory conduction and maintaining axonal electrical stability [20]. Beyond their myelinating role, OLs provide essential metabolic support to axons, a function increasingly recognized as critical for long-term axonal integrity and neuronal function [156]. Although AD has traditionally been viewed as a disorder driven primarily by neuronal Aβ production, recent studies have brought attention to glial contributions to disease pathogenesis. Increasing evidence suggests that OLs dysfunction and myelin disruption may contribute to cognitive decline and disease progression in AD [157–160]. Extensive myelin degeneration has been linked to impairments in neuronal function and cognitive performance [161]. Consistent with this, treatment with the promyelinating agent clemastine promotes oligodendrocyte precursor cell (OPC) proliferation and differentiation, enhances myelin formation, reduces Aβ deposition, and improves cognitive performance in APP/PS1 transgenic mice [161].

In addition to their vulnerability in AD, OLs have also been identified as a cellular source of Aβ. Human OLs produce Aβ and are prone to form soluble aggregates [162]. In vivo studies by Sasmita et al. indicated that OLs actively participate in amyloid plaque formation, implicating them as contributors to AD pathology [163]. Using conditional Bace1 knockout strategies in an APP knock-in background, these studies quantified the relative contributions of excitatory neurons (ExNs) and OLs to amyloid deposition. Ablation of Bace1 in ExNs resulted in a > 95% reduction in plaque burden at six months of age, whereas OL-specific Bace1 deletion led to a ~ 25% reduction in plaque number across cortical regions [163]. This effect was particularly pronounced in layers V/VI of the retrosplenial cortex compared with layers II/III, with similar reductions (20–30%) observed in the hippocampal CA1 region and corpus callosum [163]. These findings establish ExNs as the dominant source of plaque-forming Aβ, while also highlighting a meaningful contribution from OLs. Notably, substantial plaque deposition re-emerged in 12-month-old ExN-specific Bace1 knockout mice, suggesting that Aβ derived from non-neuronal populations becomes increasingly relevant with disease progression [163]. Although the overall contribution of OL-derived Aβ to plaque burden is modest, studies indicate that it may still exert pathogenic effects.

Although Aβ immunotherapy has been extensively studied in the context of neuronal and glial responses, its effects on OLs’ function and myelination remain largely unexplored, representing a notable gap in our understanding. This gap is particularly relevant given emerging evidence that OLs not only contribute to Aβ production but also play key roles in myelin integrity and neuronal network function. Interventions targeting OLs have shown therapeutic potential in AD models. For example, senolytic approaches that eliminate senescent oligodendrocytes in plaque-associated regions attenuate neuroinflammation, reduce Aβ deposition, and improve cognitive performance [164]. Although these strategies are not based on Aβ immunotherapy, they highlight the functional importance of OLs in modulating disease progression and therapeutic outcomes.

Collectively, these findings suggest that OLs may play a more significant role in Aβ homeostasis and pathology in the AD brain than previously recognized. This possibility is particularly relevant given that OLs can contribute to Aβ production [163] and are integral to myelin integrity [161] and neuronal network function, both of which are closely linked to cognitive performance. In addition, recent preclinical studies have shown that Aβ antibodies target not only amyloid plaques but also distinct brain cells and vessels, including OLs containing intracellular Aβ [11]. In principle, antibody uptake by OLs could influence intracellular Aβ handling or other OL-associated functions, thereby extending the effects of passive Aβ immunotherapy beyond extracellular plaque removal. Although the functional consequences of Aβ antibody uptake in OLs remain unclear, these findings raise the possibility that OLs may participate in cellular responses to passive Aβ immunotherapy. At present, the contribution of OLs to Aβ immunotherapy remains insufficiently defined. Future studies will be required to determine whether Aβ antibody interactions with OLs influence intracellular Aβ handling, OL function, myelin integrity, or cognitive outcomes during immunotherapy. Such studies may also clarify whether targeting OL-associated intracellular Aβ represents a previously underappreciated mechanism contributing to the therapeutic effects of Aβ antibodies.

### Perivascular macrophages (PVMs)

PVMs are a specialized population of resident macrophages strategically positioned within the perivascular spaces surrounding penetrating arterioles and venules in the brain [165–167]. Located at the neurovascular interface, PVMs contribute to CNS homeostasis by mediating immune surveillance, clearing metabolic waste, and modulating neuroinflammatory responses under both physiological and pathological conditions [168–170]. Accumulating evidence suggests that PVMs play a protective role in AD through their intrinsic capacity to recognize, engulf, and degrade Aβ aggregates [165, 171]. By facilitating perivascular Aβ clearance, PVMs may limit plaque accumulation and influence disease progression [172]. Evidence for this role comes from studies using the PVMs depletion approaches. In the 5xFAD mouse model, intracerebroventricular administration of liposome-encapsulated clodronate, which selectively ablates PVMs, resulted in a pronounced increase in amyloid plaque burden, particularly in the cerebral cortex and amygdala, regions that are critical for cognitive and emotional processing [173]. Similarly, in TgCRND8 mice, depletion of PVMs markedly exacerbated CAA, as reflected by increased numbers of thioflavin S–positive, amyloid-laden vessels and enhanced vascular deposition of aggregated Aβ42. These findings indicate that PVMs are especially important for restraining vascular amyloid accumulation [174].

Beyond animal models, emerging human data further support a role for PVMs in AD pathogenesis. Single-cell transcriptomic analyses combined with Mendelian randomization have identified several genes, Interferon Gamma Receptor 1 (IFNGR1), Kelch-Like Family Member 5 (KLHL5), NUMB Endocytic Adaptor Protein (NUMB), and WD Repeat and FYVE Domain Containing 4 (WDFY4), that are significantly associated with AD risk and are linked to immune regulation, intracellular trafficking, and inflammatory signaling pathways relevant to PVMs function [175]. Importantly, direct evidence linking PVMs to passive Aβ immunotherapy has recently emerged. Taylor et al. indicated that Aβ immunotherapy induces activation of PVMs together with recruitment of peripheral monocytes in AD mouse models [21]. Notably, this response was associated with increased vascular permeability and cerebral microhemorrhages consistent with ARIA, suggesting that PVMs may contribute not only to vascular Aβ clearance but also to neurovascular inflammatory responses during immunotherapy [21]. Given their localization within perivascular spaces, where circulating antibodies, vascular Aβ, and clearance pathways converge, PVMs are well positioned to influence the handling of antibody–Aβ complexes, regulate perivascular clearance, and modulate vascular responses to immunotherapy. Together, these observations support a role for PVMs in passive Aβ immunotherapy. In addition to their established function in vascular Aβ clearance, PVMs may influence the handling of antibody–Aβ complexes and contribute to ARIA-associated neurovascular responses during treatment. However, the precise mechanisms by which PVMs regulate therapeutic efficacy and vascular toxicity remain incompletely understood.

### Vascular and meningeal lymphatic responses to passive Aβ immunotherapy

#### Cerebrovascular effects and vascular adverse events

In postmortem brain tissue from individuals with Down syndrome, lecanemab was shown to bind not only parenchymal amyloid plaques but also vascular Aβ deposits associated with CAA [176]. These findings indicate that vascular amyloid represents an important target of anti-Aβ antibodies and support the concept that antibody engagement of CAA may contribute to vascular responses observed during Aβ immunotherapy [176]. Aβ immunotherapies robustly enhance cerebral amyloid clearance, yet vascular-related adverse effects consistently accompany them. CAA, defined by the accumulation of Aβ within cerebral vessel walls, compromises vascular integrity and function, with Aβ1–40 representing the predominant isoform [177]. In CAA, antibody engagement of vascular Aβ may alter vessel stability and contribute to treatment-related vascular adverse events. Early preclinical evidence came from APP23 mice, in which passive Aβ immunization reduced parenchymal amyloid burden but significantly increased the incidence and severity of CAA-associated hemorrhages, particularly in vessels containing amyloid deposits [39]. A major complication observed across multiple clinical trials is ARIA [178]. Neuroimaging distinguishes two principal forms of ARIA. ARIA-E presents as vasogenic edema, whereas ARIA-H is characterized by hemosiderin deposition, typically manifesting as cerebral microhemorrhages or superficial siderosis [179]. The incidence of ARIA appears to be dose-dependent. In the Phase II/III DIAN-TU-001 trial, 19% of patients treated with gantenerumab developed ARIA-E [180]. In the EMERGE and ENGAGE Phase III trials, 41% of participants receiving high-dose aducanumab experienced ARIA, with ARIA-E occurring in 35% and microhemorrhages or superficial siderosis in 19% and 15% of patients, respectively [181]. ARIA incidence has also been reported with other Aβ antibodies, although the frequency varies across therapeutic agents and dosing regimens. In the Phase III lecanemab trial (NCT03887455), ARIA-E occurred in 12.6% of participants [8]. Mechanistically, one proposed explanation for ARIA is that antibody binding to vascular Aβ compromises vessel wall integrity and increases vascular permeability [178, 182]. Supporting this view, Wilcock and colleagues reported that active and passive Aβ immunization induced marked upregulation of matrix metalloproteinase-2 (MMP-2) and MMP-9 in animals exhibiting pronounced microhemorrhages, implicating MMP-mediated vascular degradation in ARIA pathogenesis [183]. While vascular Aβ removal and MMP-mediated vessel wall disruption provide one explanation for ARIA, other observations suggest that changes in the spatial distribution of vascular Aβ during immunotherapy may also contribute. Postmortem analyses following Aβ immunization have suggested that amyloid removal from the brain parenchyma may be accompanied by increased vascular amyloid deposition [184]. Based on these observations, it has been proposed that immunotherapy may promote redistribution of Aβ species toward cerebral vessel walls, potentially increasing vascular stress and contributing to vessel fragility. However, the extent to which such vascular redistribution directly contributes to ARIA remains incompletely understood. Importantly, recent studies suggest that vascular adverse events may not be solely dependent on Aβ clearance itself. In a phase II trial, the TREM2 agonistic antibody AL002 showed evidence of target engagement and microglial activation but did not significantly reduce brain amyloid burden or slow clinical progression. Nevertheless, ARIA was observed during treatment, raising the possibility that immune activation within the neurovascular unit may contribute to vascular dysfunction independently of substantial amyloid removal [185]. These findings suggest that ARIA arises from a multifactorial process in which antibody engagement, immune activation, and vascular vulnerability interact.

In summary, while Aβ immunotherapies have proven effective in reducing brain Aβ burden, their vascular side effects, particularly ARIA, remain a major safety concern. Importantly, even if ARIA is not strictly dependent on Aβ clearance, antibody–target interactions within the neurovascular unit may still trigger downstream immune and vascular responses. Future work should therefore dissect the relative contributions of vascular amyloid clearance and immune-mediated vascular responses to ARIA pathogenesis, which may facilitate the development of safer immunotherapeutic strategies.

#### Meningeal lymphatic vessels (mLVs)

Meningeal lymphatic vessels (mLVs) form a lymphatic network embedded within the meninges and serve as a major outflow pathway for CSF, interstitial solutes, and cellular debris from the CNS to the deep cervical lymph nodes (dCLNs) [186, 187]. Functionally linked to the glymphatic system, mLVs cooperate in the clearance of soluble proteins, metabolites, and waste products from the brain [188–190]. Ablation of mLVs markedly reduces the transport of macromolecular tracers, such as OVA–647, from the cisterna magna to the dCLNs, supporting their essential role in removing large molecules from the brain interstitium [191]. Growing evidence implicates mLV dysfunction in the progression of AD. In 13–14-month-old 5xFAD mice, deterioration of dorsal meningeal lymphatics is associated with extensive Aβ accumulation throughout the meninges, including deposition along blood vessels and lymphatic structures [23]. Similarly, impaired lymphatic drainage in APP/PS1 mice leads to the accumulation of Aβ aggregates within dural lymphatic vessels and exacerbates parenchymal plaque burden [192]. Notably, near-infrared stimulation has been shown to enhance mLV function in 5xFAD mice, resulting in increased Aβ clearance from the hippocampus and prefrontal cortex and improved performance in novel object recognition and Y-maze tasks [193].

MLVs have emerged as important regulators of the response to Aβ immunotherapy. Ablation of mLVs in 5xFAD mice attenuates the plaque-clearing effects of aducanumab and mAb158 (murine version of lecanemab), leading to increased amyloid burden and more abundant LAMP1-positive dystrophic neurites compared with mice with intact lymphatic drainage [23]. Mechanistic studies suggest that impaired mLV function reduces perivascular CSF influx, thereby limiting antibody access to parenchymal Aβ. Conversely, enhancement of lymphatic function, for example, through intracisternal administration of mAb158 combined with vascular endothelial growth factor-C (VEGF-C), significantly increases plaque removal, indicating that mLVs influence antibody distribution within the brain and treatment efficacy [23].

Overall, these observations indicate that mLVs are not merely passive drainage pathways but an essential component of the neurovascular–lymphatic clearance system that governs antibody access, distribution, and therapeutic efficacy in Aβ immunotherapy. By regulating CSF dynamics and perivascular transport, mLVs critically influence the extent to which peripherally administered antibodies can penetrate the brain and engage parenchymal Aβ. Targeting mLVs, either to improve fluid transport or to enhance clearance capacity, may therefore represent a promising strategy to enhance the efficacy of Aβ immunotherapies. Future studies should define how mLVs regulate the spatial distribution of therapeutic antibodies within the CSF, perivascular spaces, and brain parenchyma, and determine whether age-related lymphatic dysfunction contributes to interindividual variability in treatment response. Addressing these questions may facilitate the development of strategies that combine lymphatic enhancement with passive Aβ immunotherapy to improve therapeutic efficacy.

### Peripheral monocytes

#### Role of peripheral monocytes in AD pathogenesis

Peripheral monocytes are increasingly recognized as active modulators of AD pathology [194]. In patients with AD, circulating monocytes show increased infiltration into the CNS but exhibit reduced Aβ-clearing capacity and a shift toward a pro-inflammatory phenotype [195]. Functionally, activated monocytes promote astrocyte reactivity, induce neuronal apoptosis, and secrete inflammatory mediators such as interleukin-1β (IL-1β) and C-C motif chemokine ligand 3 (CCL3), thereby amplifying neuroinflammatory signaling and recruiting additional immune cells to affected regions [195]. Peripheral monocytes also participate in systemic Aβ homeostasis. Approximately 40–60% of Aβ generated in the brain ultimately enters the circulation, highlighting the importance of peripheral clearance pathways in regulating overall Aβ burden [196, 197]. At the vascular interface, they remove Aβ from the luminal side of cerebral vessels [198]. In addition, infiltrating monocytes can enter the brain parenchyma, interact with amyloid plaques, and promote their degradation [199].

Given their multifaceted roles in Aβ clearance and neuroinflammation, peripheral monocytes are increasingly recognized as a heterogeneous population with distinct functional subsets, differing in their capacity for Aβ binding, transport, and clearance. Distinct Aβ-responsive monocyte subsets have been identified. A CD14⁺/CD16⁺ population with strong Aβ-binding and phagocytic capacity is increased in individuals with mild cognitive impairment (MCI) and AD and has been proposed to traffic between the CNS and the periphery, suggesting a bidirectional Aβ transport axis [200]. Meanwhile, circulating Aβ aggregates in MCI patients activate CD18⁺ monocytes via complement receptor 4, enhancing phagocytosis and lysosomal degradation [201]. Notably, both the abundance and functional capacity of these Aβ-reactive monocytes decline with disease progression, correlating with increasing cerebral Aβ burden and worsening cognitive performance [201, 202].

### Peripheral monocyte responses to Aβ immunotherapy

Peripheral monocytes and monocyte-derived macrophages have emerged as potential modulators of the response to Aβ immunotherapy, although direct evidence in humans remains limited. Preclinical studies provide important mechanistic insights into how antibody treatment engages peripheral myeloid cells. In AD mouse models, administration of Aβ antibodies such as 3D6 promotes the formation of antibody–Aβ immune complexes at sites of vascular amyloid deposition and activates Cluster of Differentiation 169 (CD169)⁺ perivascular macrophages, accompanied by recruitment of circulating inflammatory monocytes [21]. Importantly, this recruitment was linked to increased vascular permeability and cerebral microhemorrhages exhibiting ARIA-like vascular features, suggesting a potential connection between Aβ immunotherapy-induced vascular injury and peripheral myeloid cell responses [21].

Antibody binding to Aβ enhances Fcγ receptor–mediated recognition and uptake by myeloid cells [203, 204], suggesting a mechanism by which peripheral monocytes and monocyte-derived macrophages may participate in antibody-dependent Aβ clearance. In addition, peripheral myeloid cells have been shown to infiltrate the brain and contribute to amyloid clearance under certain conditions [205–207], although the specific contribution of circulating monocytes following Aβ antibody treatment remains to be fully defined. Beyond these mechanisms, peripheral monocytes may influence Aβ immunotherapy through their role in systemic Aβ dynamics. Antibody binding may alter the distribution of Aβ between central and peripheral compartments, consistent with the peripheral sink hypothesis [37], and circulating monocytes may participate in the uptake and processing of antibody–Aβ complexes in the periphery, thereby indirectly shaping brain Aβ burden. Importantly, peripheral monocytes are a heterogeneous population with distinct functional subsets [208]. Inflammatory C–C chemokine receptor type 2 (CCR2)⁺ monocytes are preferentially recruited to sites of vascular amyloid and are associated with enhanced inflammatory responses [206], whereas other monocyte subsets, particularly patrolling monocytes, contribute to vascular immune surveillance and debris clearance under homeostatic conditions [209]. The balance between these subsets may therefore influence whether antibody treatment results in beneficial Aβ removal or detrimental vascular inflammation.

Together, these observations suggest that peripheral monocytes are not merely recruited effectors but may function as dynamic regulators of both central and systemic responses to Aβ immunotherapy. An important unresolved question is whether distinct monocyte subsets differentially contribute to the beneficial and detrimental effects of Aβ immunotherapy. Defining how antibody treatment influences monocyte recruitment, phenotype, and function may help identify strategies that enhance Aβ clearance while minimizing vascular inflammation and ARIA-related pathology.

### Integrated multicellular clearance network

**Fig. 4:**
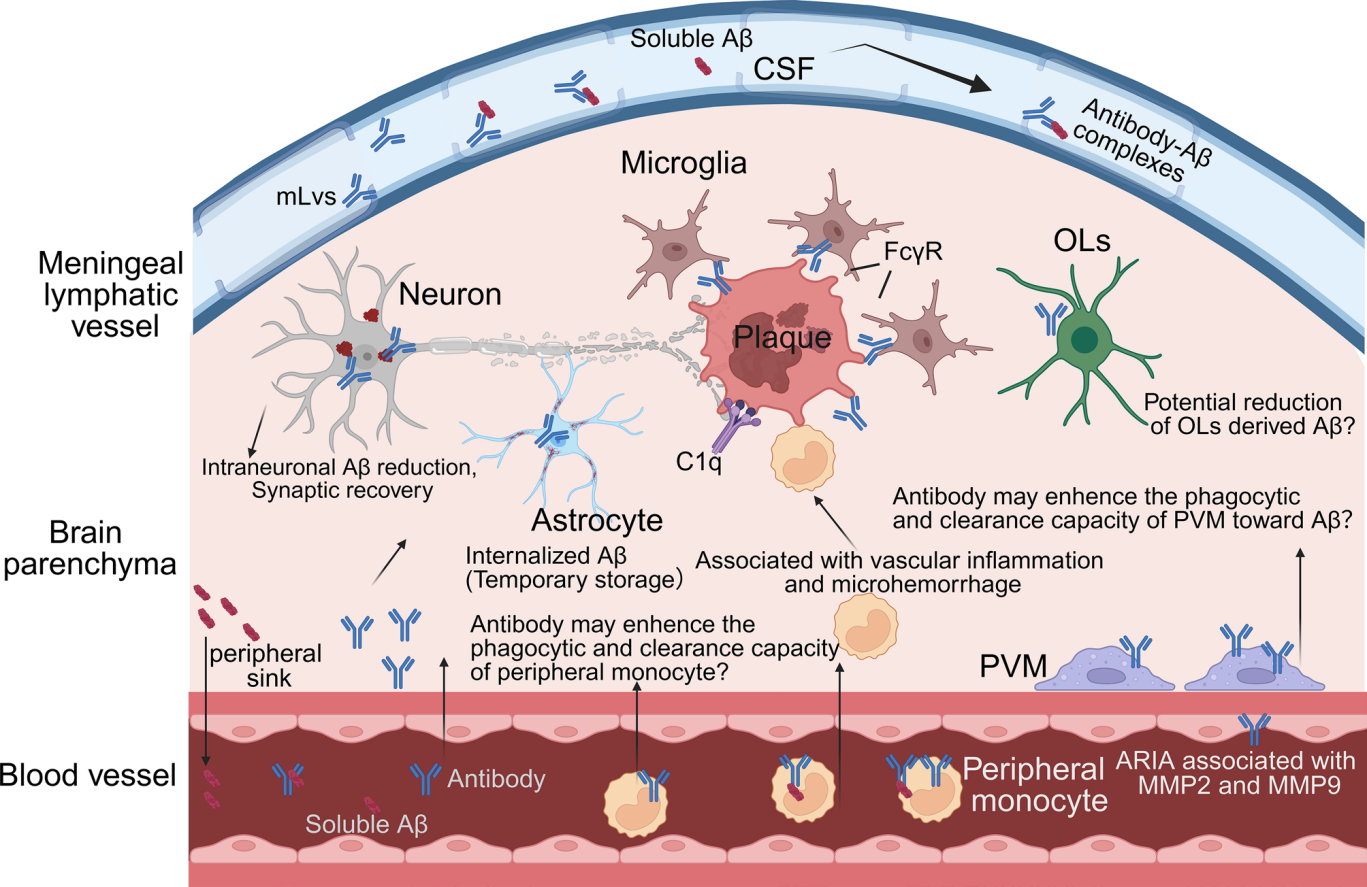
Multicellular and spatially organized mechanisms of Aβ antibodies in the brain. Peripherally administered Aβ antibodies circulate within the bloodstream and enter the central nervous system (CNS). Within the vascular compartment, Aβ antibodies can bind soluble Aβ in the circulation, where circulating monocytes contribute to peripheral Aβ uptake and clearance. In AD, subsets of peripheral monocytes can migrate across the blood–brain barrier (BBB) and accumulate in the brain parenchyma, where they localize near amyloid plaques and may participate in plaque-associated immune responses. Antibody–Aβ interactions in the circulation have also been proposed to promote redistribution of Aβ from the brain to the periphery, consistent with the *peripheral sink* hypothesis. According to this hypothesis, Aβ antibody binding to circulating Aβ shifts the equilibrium across the BBB, favoring efflux of soluble Aβ from the brain into the vascular compartment. Within the brain, Aβ antibodies distribute across multiple anatomical compartments, including cerebrospinal fluid (CSF), perivascular spaces, and brain parenchyma. Aβ antibodies are not restricted to extracellular plaques but can be detected within neurons and glial cells. In neurons, Aβ antibody internalization has been shown to reduce intraneuronal Aβ (iAβ) and is associated with recovery of synaptic function in AD mouse models. Microglia interact with antibody–Aβ complexes through FcγR and complement components such as C1q, contributing to plaque-associated clearance. Astrocytes can internalize Aβ and may interact with antibody-bound Aβ species, but available evidence suggests limited degradative capacity, consistent with a transient intracellular accumulation rather than sustained clearance. Aβ antibodies have also been detected in oligodendrocytes (OLs) and perivascular macrophages (PVMs), although the functional consequences of these interactions remain incompletely defined. In the cerebrovasculature, amyloid-related imaging abnormalities (ARIA) are associated with matrix metalloproteinases (MMP-2 and MMP-9) and immune cell recruitment. Antibody–Aβ complexes in the CSF may be cleared via meningeal lymphatic vessels (mLVs), linking central antibody distribution to peripheral drainage pathways. Together, this schematic illustrates that passive Aβ immunotherapy operates within a multicellular and anatomically organized clearance network, in which Aβ antibody distribution and cellular interactions critically regulate both therapeutic efficacy and vascular risk

Collectively, these findings indicate that the effects of passive Aβ immunotherapy extend well beyond neurons, astrocytes, and microglia, engaging a broader cellular and vascular clearance network that includes oligodendrocytes, perivascular macrophages, peripheral monocytes, and meningeal lymphatic pathways, as summarized in Fig. 4. These components interact to influence not only the efficiency with which antibodies access and remove parenchymal Aβ, but also the downstream redistribution of amyloid along vascular and perivascular compartments. Disruption or imbalance within this network may contribute to both limited therapeutic efficacy and vascular adverse events. Together, these observations support the view that passive Aβ immunotherapy operates within a multicellular and spatially organized clearance network, in which antibody distribution and cell-specific interactions are likely to be important determinants of both therapeutic efficacy and treatment-associated toxicity.

## Conclusion

It has been demonstrated that passive Aβ immunotherapy has the capacity to slow the rate of cognitive impairment, although clinical benefits remain variable [182, 210]. Meanwhile, safety concerns (particularly ARIA) continue to present a major obstacle to clinical translation [8, 82, 181]. To date, both preclinical and clinical studies have focused predominantly on plaque reduction. However, the evidence reviewed here suggests that therapeutic efficacy and safety are not determined solely by plaque removal. Instead, Aβ antibodies engage a multicellular and spatially organized network involving neurons, glial cells, perivascular macrophages, peripheral immune cells, and meningeal lymphatic pathways, which collectively influence Aβ clearance, neuroinflammatory responses, vascular complications, and ultimately treatment outcomes. In particular, intraneuronal Aβ and cell-type–specific responses to Aβ antibodies emerge as potentially important but insufficiently explored determinants of therapeutic efficacy and safety.

Although these findings broaden our understanding of how Aβ immunotherapy operates within the brain, they also underscore the need for therapeutic strategies that improve efficacy while minimizing treatment-associated adverse events. One important direction is to enhance antibody delivery and target accessibility within the CNS. ATV engineered to target the TfR have been shown to enhance brain penetration of Aβ antibodies in animal models [94], supporting the subsequent development of Brain shuttle-based Aβ antibodies such as Trontinemab. These approaches illustrate how improving antibody delivery and target accessibility may enhance therapeutic efficacy while potentially reducing dose-related adverse events. In parallel, subcutaneous formulations of Aβ antibodies are being explored as an alternative route of administration. Compared with intravenous infusion, subcutaneous delivery may improve treatment convenience and reduce the burden associated with repeated infusions. Ongoing clinical evaluation of subcutaneous formulations for antibodies such as remternetug may further expand the accessibility and practicality of passive Aβ immunotherapy [87].

While optimizing antibody delivery may improve the performance of existing Aβ-directed therapies, additional gains in efficacy may require targeting multiple pathological pathways simultaneously. A second direction is to move beyond plaque-centered approaches toward interventions that address multiple pathological and cellular mechanisms simultaneously. In this context, combination immunotherapy targeting both Aβ and tau pathology has emerged as a particularly promising strategy. An ongoing platform trial (NCT06957418) is evaluating tau-directed therapies alone or in combination with the anti-Aβ monoclonal antibody donanemab, representing one of the first clinical efforts to simultaneously target both amyloid and tau pathologies in sporadic AD [211]. This strategy reflects a growing recognition that effective disease modification may require intervention at multiple pathological levels rather than focusing exclusively on amyloid removal.

Beyond targeting pathological proteins directly, emerging immunotherapeutic strategies are also beginning to focus on immune regulatory pathways. In particular, emerging targets such as TREM2 further highlight the importance of modulating microglial and neuroimmune responses as part of future therapeutic strategies [212]. Together, these advances suggest that the future of AD immunotherapy may extend beyond extracellular plaque removal toward integrated strategies that optimize antibody distribution within the brain, engage multicellular clearance and immune networks, and simultaneously target multiple pathological processes that drive disease progression.

## Data Availability

No datasets were generated or analysed during the current study.

## Abbreviations

Aβ: Amyloid-beta
AD: Alzheimer’s disease
ApoE: Apolipoprotein E
APP: Amyloid precursor protein
ARIA: Amyloid-related imaging abnormalities
ATV: Antibody transport vehicle
BBB: Blood–brain barrier
CAA: Cerebral amyloid angiopathy
CAR-As: Chimeric antigen receptor astrocytes
CD68: Cluster of differentiation 68
CD169: Cluster of differentiation 169
CL: Centiloid
CNS: Central nervous system
CSF: Cerebrospinal fluid
DAM: Disease-associated microglia
ExNs: Excitatory neurons
FcγR: Fc gamma receptor
GFAP: Glial fibrillary acidic protein
iAβ: Intraneuronal amyloid-beta
IFNGR1: Interferon gamma receptor 1
IgG: Immunoglobulin G
IL-1β: Interleukin-1 beta
Itgax: Integrin alpha X
KLHL5: Kelch-like family member 5
LRP1: Low-density lipoprotein receptor-related protein 1
mLVs: Meningeal lymphatic vessels
MMP-2: Matrix metalloproteinase-2
MMP-9: Matrix metalloproteinase-9
NUMB: NUMB endocytic adaptor protein
OLs: Oligodendrocytes
OPCs: Oligodendrocyte precursor cells
PE: Positron emission tomography
PVMs: Perivascular macrophages
PVS: Perivascualr space
TfR: Transferrin receptor
TfR1: Transferrin receptor 1
TREM2: Triggering receptor expressed on myeloid cells 2
TRIM21: Tripartite motif-containing protein 21
VEGF-C: Vascular endothelial growth factor C
WDFY4: WD repeat and FYVE domain containing 4
